# Medical Papers in Public Libraries

**Published:** 1916-02-12

**Authors:** 


*To the Editor of* The Hospital.

#### Medical Papers in Public Libraries.

*To the Editor of* The Hospital.

Sir,—It is not often that a newspaper dealing with medicine and its allied subjects finds its way into the public library, but the rule is proved by the exception which is to be found in the case of the Worthing Public Library, where there is weekly provided for readers a copy of the current issue of The Hospital.—Yours faithfully, B. R.

February 5, 1916.

